# *Phaeodactylum tricornutum* as a Chassis: Insights into Its Potential, Challenges, and Perspectives

**DOI:** 10.3390/md24020079

**Published:** 2026-02-13

**Authors:** Sen Wang, Yunuo Hao, Tengsheng Qiao, Ruihao Zhang, Deliang Yu, Hailiang Wang, Yongliang Liu, Yuhao Sun, Di Xu, Xiaojin Song, Kehou Pan

**Affiliations:** 1 Laoshan Laboratory, Qingdao 266237, China; senwang2012@163.com (S.W.); 18314331059@163.com (T.Q.); qdzhangruihao@163.com (R.Z.); ydeliang1919@163.com (D.Y.); whl846130@126.com (H.W.); lyliocas@163.com (Y.L.); 18669884128@163.com (Y.S.); 2 Key Laboratory of Marine Genetics and Breeding, Ministry of Education, College of Marine Life Sciences, Ocean University of China, Qingdao 266003, China; hynuo@foxmail.com (Y.H.); dixu@ouc.edu.cn (D.X.)

**Keywords:** *Phaeodactylum tricornutum*, photosynthetic chassis, strain improvement, synthetic production, cultivation condition

## Abstract

*Phaeodactylum tricornutum* is one of the most well-characterized microalgae and serves as a pivotal model diatom in global carbon fixation and the mediation of biogeochemical cycling of essential nutrients. Over the past few decades, the availability of a complete genome assembly, coupled with the development of robust DNA manipulation tools and efficient DNA delivery methodologies, has established *P. tricornutum* as a promising photosynthetic chassis for the sustainable bioproduction of high-value compounds, including fucoxanthin and eicosapentaenoic acid (EPA). This review systematically summarizes the research progress in the strain improvement toolkit of *P. tricornutum*, encompassing both genetic and non-genetic engineering strategies. It elaborates on the types and applications of its representative bioactive products, as well as the molecular mechanisms underlying key synthetic pathways. Additionally, this work synthesizes the research findings on the optimization of critical cultivation conditions (e.g., light, temperature, and nutrient composition) that modulate the growth and product synthesis of *P. tricornutum*. On this basis, the challenges encountered by *P. tricornutum* in industrial applications are proposed for further discussion, aiming to provide a reference for in-depth exploration of related research directions and facilitate the expansion of its application scope in the field of biomanufacturing.

## 1. Introduction

Diatoms are the most abundant group of marine and freshwater phytoplankton that contribute more than 20% of global carbon fixation and mediate biogeochemical transfer of important nutrients, such as nitrogen, carbon, and silicon [1]. They are single-celled photosynthetic algae (although several chain-forming species exist, including members of the genera *Skeletonema* [2,3], *Chaetoceros* [4,5], and *Thalassiosira* [5,6,7]) and represent a highly diverse group with nearly 100,000 distinct species [8]. As a model diatom, *Phaeodactylum tricornutum* is arguably the best-characterized diatom to date. Its utility in elucidating the functions of genes, proteins, and metabolites in diatoms is enhanced by its easy cultivation along with reproducible genetic transformation and cryopreservation [9,10,11].

Synthetic biology is an emerging interdisciplinary field, broadly defined by the pursuit of designing, synthesizing, and “booting up” DNA within a target host organism to construct functional biological systems or produce desired products. Among photoautotrophic species, the diatom *P. tricornutum* has been successful in establishing genetic engineering approaches, including genome editing and gene expression, and it is commercially viable for large-scale cultivation, making it a promising candidate as a suitable chassis for sustainable bioproduction. To date, it is known for accumulating a diverse range of marketable bioproducts, including lipid-based nutraceuticals (e.g., eicosapentaenoic acid (EPA) [12]), anti-inflammatory agents (e.g., brassicosterol [13]), anti-tumor chemicals (e.g., betulin [13]), biofuels (e.g., triacylglycerols (TAG) [14]), bioplastics (e.g., polyhydroxybutyrate [15]), and recombinant monoclonal antibodies (e.g., human IgGαHBsAg [16]). It exhibits considerable potential as a chassis for sustainable bioproduction. However, compared with traditional chassis, such as *Escherichia coli* and *Saccharomyces cerevisiae*, it still has many application limitations that need to be addressed. These drawbacks include the poor economic viability of its production processes, which stem from low biomass and product titers, as well as suboptimal cultivation systems and low efficiency in strain engineering, which is attributed to incomplete genetic characterization and underdeveloped advanced gene-editing tools, among others. In this review, we aim to provide a comprehensive overview of *P. tricornutum*’s application potential as a photoautotrophic chassis, the key challenges limiting its industrialization, and optimization strategies (e.g., genetic engineering, metabolic regulation, and cultivation condition improvement) developed to address these bottlenecks.

## 2. Taxonomic, Morphological, and Genomic Characteristics of *P. tricornutum*

*P. tricornutum* has long served as a standard “lab rat” for research in marine planktonology. However, in the early literature on algal taxonomy and cultivation, this species was initially misidentified and classified under the genus *Nitzschia* [17]. It was not until 1958 that Lewin clarified the morphological and taxonomic characteristics distinguishing *P. tricornutum* from *Nitzschia closterium* (Ehr.) W.Sm. He further proposed establishing a new suborder, Phaeodactylineae, within the Bacillariales, with *Phaeodactylum* designated as the only known genus in this suborder [17,18]. Subsequently, molecular phylogenetic analysis provided further evidence to confirm that *Phaeodactylum* belongs to the Naviculales order [19]. Most recently, comparative genomic analysis further underscored the distinctiveness of *P. tricornutum*. The study of *N. closterium f. minutissima* and *P. tricornutum* revealed that the two species share only 26 gene families, providing molecular-level confirmation of their differences [20]. With advancements in molecular systematic techniques (e.g., whole-genome sequencing and phylogenomics), the classification system of diatoms continues to be refined. Currently, the hierarchical taxonomic status of “Naviculales–Phaeodactylaceae—Phaeodactylum” is widely recognized by the international algal taxonomy community. Beyond its classification, *P. tricornutum* has demonstrated remarkable ecological and taxonomic consistency: over the past few decades, isolates of this species have been reported and cultured from diverse global locations, and genetic and morphological evidence consistently confirm these isolates belong to the same species [21].

**Figure 1:**
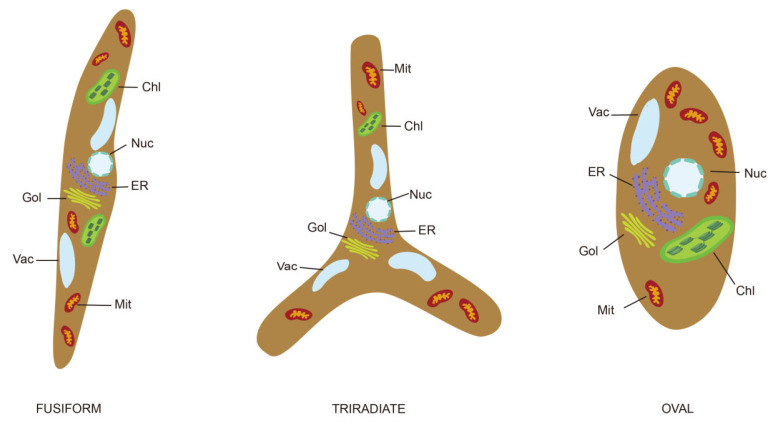
The three major morphotypes of *P. tricornutum*. Chl: chloroplast; Nuc: nucleus; ER: endoplasmic reticulum; Gol: Golgi complex; Vac: vacuole; Mit: mitochondrion.

Most diatoms possess a unique box-shaped cell wall termed the frustule, a structure primarily composed of silica, with minor contributions from proteins and carbohydrates [22]. Compared with other diatom species, *P. tricornutum* is considered an “atypical diatom”, primarily due to its polymorphic morphotypes [17] and the morphotype-specific distribution of siliceous structures [23]. It exists in three major morphotypes: oval, fusiform, and triradiate (Figure 1). Only the oval morphotype contains organized silicified frustules, whereas the fusiform and triradiate morphotypes show poor silicification or even lack distinct silicified frustules entirely [24]. Moreover, morphological shifts between these three types are observable under specific culture conditions [25], suggesting this plasticity may be an adaptive response to environmental fluctuations [21]. Specifically, such morphotype changes are one plausible phenotypic alteration associated with transitions between planktonic and benthic habitats [26].

*P. tricornutum* exhibits metabolic flexibility across photoautotrophic and mixotrophic growth modes [27]. Autotrophic growth synthesizes organic matter using CO_2_ and light energy, enabling simultaneous carbon sequestration and sustainable biomass production. In addition, it can metabolize organic carbon in the presence of light; this mode, which involves the simultaneous utilization of photosynthesis and respiration, is of particular interest for understanding how these two major energy systems interact with one another. Most strains of *P. tricornutum* could use glycerol and urea as organic carbon and nitrogen sources, and biomass could reach 3~15 g/L under mixotrophic mode [28]. Notably, *P. tricornutum* is naturally an obligate photoautotroph primarily due to its lack of endogenous transporters for efficient organic carbon uptake [29]. Heterotrophic growth can be achieved by introducing a single gene, which encodes a glucose transporter protein (Glut1) [30,31,32].

The complete genome sequence of *P. tricornutum* was first reported in 2008 [33]. It spans approximately 27.4 megabases (Mb), with 33 predicted chromosomes and 10,402 annotated genes. More recently, third-generation sequencing has achieved telomere-to-telomere coverage of the 25 chromosomes present in the *P. tricornutum* nucleus [34], providing additional information for this species. Additionally, the chloroplast and mitochondrial genomes have also been published [35,36]. As the report indicated, its chloroplast genome is compact, with small intergenic regions and no introns, thereby rendering it a promising target for genetic engineering.

## 3. Strain Improvement Toolkit for *P. tricornutum*

**Figure 2:**
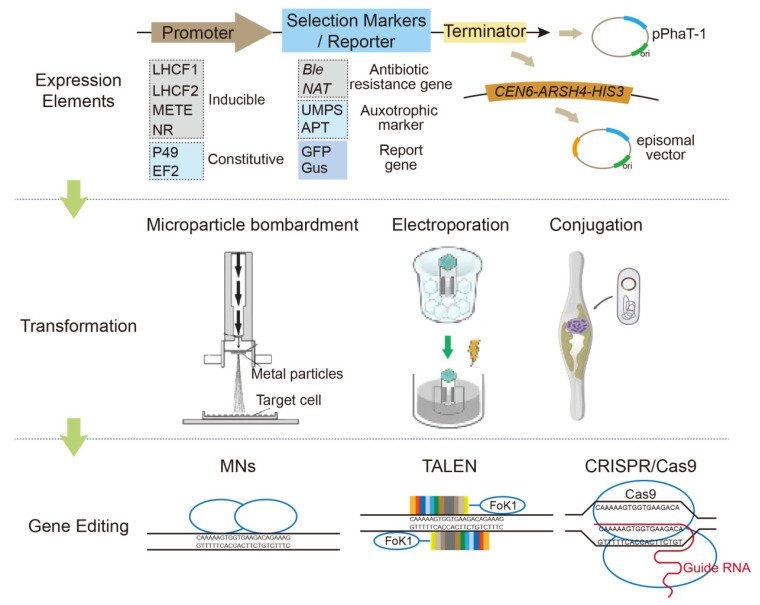
Strain improvement toolkit for *P. tricornutum*. Core components consist of gene expression elements (widely utilized promoters, selectable markers, reporter genes, terminators, and plasmid backbones), three established transformation methods (microparticle bombardment, electroporation, and conjugation), and gene editing systems (MNs, TALENs, and CRISPR/Cas9). LHCF1: the light-regulated *fcpA* promoter; LHCF2: the light-regulated *fcpB* promoter; METE: a vitamin B12-repressed methionine synthase promoter; NR: the nitrate reductase promoter; P49: the Phatr3_J49202 gene promoter; EF2: the elongation factor 2 promoter; *Ble*: bleomycin resistance gene; *NAT*: nourseothricin acetyltransferase gene; UMPS: Uridine 5’-monophosphate synthase; APT: adenine phosphoribosyltransferase; GFP: green fluorescent protein; Gus: β-glucuronidase; CEN6-ARSH4-HIS3: the yeast-derived sequence using for episomal plasmid maintenance; MN: meganucleases; TALEN: transcription activator-like effector nucleases; CRISPR/Cas9: clustered regularly interspaced short palindromic repeats system.

The genetic toolkit developed for *P. tricornutum* is one of the most advanced, and both nuclear [37] and chloroplast [38] transformations have been achieved. Specifically, the fully sequenced nucleus [33,34], chloroplast [36], and mitochondrial [35] genomes of this species provide sufficient genetic information to support potential synthetic biology applications, enabling targeted manipulation of this microalgae cell factory. Since the first genetic transformation of *P. tricornutum* was achieved by microparticle bombardment in 1996 [39], various genetic tools have been developed, including novel transformation approaches, gene expression elements (selection markers, reporter genes, promoters, and vectors), and genome-editing tools (Figure 2).

Transformation in *P. tricornutum* appears to be more reproducible and efficient than that of other diatoms, likely because its cell wall is only partially silicified and does not require absolute silicate [40]. In addition to microparticle bombardment, electroporation [41] and conjugation [42] have been successfully developed. Transformation in *P. tricornutum* is reproducible and efficient, and the ability to efficiently grow and to recover independent colonies on agar plates following transformation and appropriate selection has facilitated genetic modification of *P. tricornutum* compared to other diatoms.

To date, traditional selection strategies for genetic engineering, including antibiotic resistance genes (e.g., for zeocin, nourseothricin, and phleomycin), auxotrophic markers (e.g., 5′-monophosphate synthase (UMPS) and adenine phosphoribosyltransferase (APT)), and reporter genes (e.g., the β-glucuronidase gene (Gus) and green fluorescent protein gene (GFP)), have all proven effective for the genetic manipulation of *P. tricornutum* [43,44]. To regulate transgene expression, a variety of endogenous promoters have also been validated as effective in *P. tricornutum*. Among these, the light-regulated promoters *fcpA* (corresponding to gene LHCF1) and *fcpB* (corresponding to gene LHCF2) and the nitrate reductase (NR) promoter are the most widely used. The LHCF1 and LHCF2 promoters are constrained by light dependency and are inactive in the dark [45], whereas the NR promoter is regulated by changes in the nitrogen sources within the medium [46]. Recently, a vitamin B12-repressed methionine synthase (METE) promoter was also identified with a dynamic range > 100-fold [47]. Several constitutive promoters were identified, such as EF2 (encoding elongation factor 2) and P49 (corresponding to gene Phatr3_J49202). These constitutive promoters exhibit higher expression levels than LHCF2 and are not significantly affected by light. Furthermore, researchers have developed a promoter library for *P. tricornutum* containing various promoters with distinct transcriptional strengths, tailored to regulate gene expression [48]. In terms of gene expression vectors, several plasmids suitable for *P. tricornutum* gene editing have been developed, including pPhaT-1, p0521s, and their derivatives. The pPhaT-1 plasmid carries a zeocin resistance gene, often serves as a backbone for gene expression vectors [46,49], and is one of the most commonly used vectors in the genetic manipulation of *P. tricornutum*. The p0521s and its derivatives, such as PtGE34/35, contain a yeast-derived sequence (CEN6-ARSH4-HIS3), which endows these plasmids with the ability to replicate stably in *P. tricornutum* and maintain a copy number equivalent to that of native chromosomes [42].

Genome editing techniques serve as a cornerstone for enhancing the traits of chassis cells via genetic engineering approaches. In recent years, meganucleases (MNs) [50], transcription activator-like effector nucleases (TALENs) [50,51,52], and clustered regularly interspaced short palindromic repeat (CRISPR/Cas9) systems [51,53,54] have emerged as efficient genome-editing tools in *P. tricornutum*. Among these three tools, CRISPR/Cas9 systems have been successfully derived and optimized to enable multi-gene knockout [51,55]. Notably, the introduction of gene targeting via homologous recombination (HR) enables precise genetic modifications, specifically the knock-in of a target sequence at a predefined locus, rather than being limited to gene knockout alone. In *P. tricornutum,* both HR and nonhomologous end joining (NHEJ) pathways are present [49]. While HR-mediated gene targeting has been demonstrated in *P. tricornutum*, its efficiency remains relatively low regardless of whether nucleases are employed. For instance, a TALEN-mediated HR can achieve an efficiency of up to 27% [50,52], and CRISPR-mediated HR exhibits a biallelic HR efficiency of 17% [55]. Currently, randomly integrated chromosomal expression or the use of episomal expression remains the most common strategy for target sequence knock-in in *P. tricornutum* [56,57,58,59].

Random mutagenesis and adaptive laboratory evolution (ALE) have also been developed for the strain improvement of *P. tricornutum* (Table 1) [60,61]. Currently, various mutagens have been used to induce random mutagenesis, including non-ionizing mutagens (e.g., ultraviolet radiation (UV) and ethylmethanosulfonate (EMS)) and an ionizing mutagen (e.g., X-rays). Miller et al. [60] evaluated the mutagenic effects of UV, EMS, and X-rays on *P. tricornutum* and found that an EMS-induced mutant strain exhibited a maximum fucoxanthin content reaching 135 ± 10% of the wild-type level within 6 months post-treatment. In another study, Yi et al. [62] generated *P. tricornutum* mutants by EMS mutagenesis, and five selected mutants displayed a ≥ 33% increase in total carotenoid content compared with the wild-type strain. In fact, microorganisms can acquire beneficial phenotypes through random genetic mutations, enabling them to rapidly adapt to changing environments, which serves as the fundamental basis for ALE. During ALE, microorganisms are cultured repeatedly under specific stress conditions to elicit positive phenotypes [63]. Yi et al. [61] combined photo-oxidative stress-driven mutagenesis with 11 consecutive ALE cycles to generate a strain with a fucoxanthin content 2.1-fold higher than that of the initial strain. Su et al. [64] obtained strains capable of exhibiting growth increases of 110.4%, 46.1%, and 27.5% at pH 5.5, 6.0, and 6.5, respectively, compared with the parental wild-type strain via 16 cycles of acidic stress-based ALE. In contrast to genetic modification technology, ALE offers the advantage of concomitantly regulating multiple distinct genes without introducing exogenous genes, and numerous studies have leveraged this technique to enhance microalgal growth characteristics, stress tolerance, metabolic activity, and substrate utilization efficiency [65,66,67]. As such, it stands as a promising strategy to optimize the phenotype traits, physiological performance, and genetic stability of *P. tricornutum*. Moreover, several methods, such as fluorescence-activated cell sorting (FACS) [60], Nile red staining [61], and selectable markers [68], have been employed to accelerate the screening rate of target cells based on the desired phenotypes. However, it is important to note that mutants generated via random mutagenesis undergo reversion to the wild-type phenotype [60]; this reversion often occurs because the diploid genome retains a functional copy of the mutated gene, which can be expressed to restore the original phenotypic traits when the mutated allele is lost or silenced.

## 4. Bioproducts and Their Strain Improvement Strategy in *P. tricornutum*

To date, *P. tricornutum* has been engineered to produce a wide range of bioproducts [81,82,83]. These include native compounds such as fucoxanthin and eicosapentaenoic acid (EPA), as well as heterologous products like recombinant proteins and bioplastics. These applications span the pharmaceutical, nutraceutical and industrial sectors.

### 4.1. Fucoxanthin

**Figure 3:**
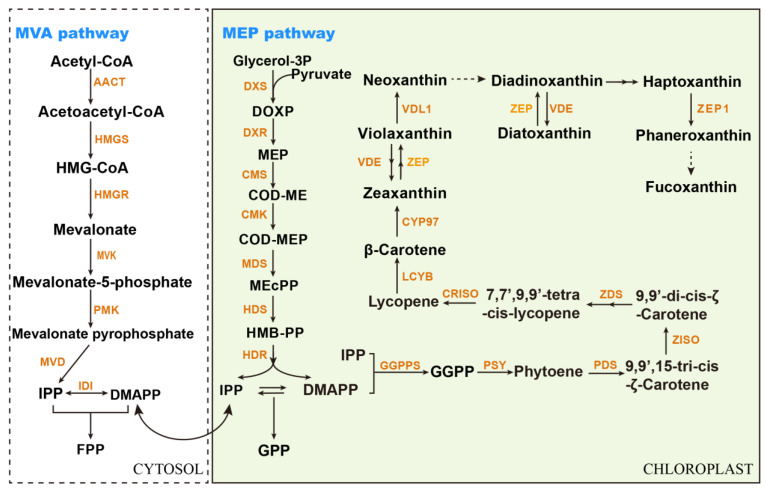
Biosynthetic pathway of fucoxanthin in *P. tricornutum*. MVA: mevalonate; MEP: 2-C-methylerythritol 4-phosphate; AACT: acetoacetyl-CoA thiolase; HMGS: 3-hydroxy-3-methylglutaryl-CoA synthase; HMGR: 3-hydroxy-3-methylglutaryl-CoA synthase; MVK: mevalonate kinase; PMK:phosphomevalonate kinase; MVD: diphospho-mevalonate decarboxylase; IDI: isopentenyl diphosphate Δ-isomerase; DXS: 1-deoxy-D-xylulose 5-phosphate synthase; DXR: 1-deoxy-D-xylulose 5-phosphate reducto-isomerase; CMS: 2-C-methyl-d-erythritol 4-phosphate cytidylyltransferase; CMK: 4-diphosphocytidyl-2-C-methyl-D-erythritol kinase; MDS: 2-C-methyl-D-erythritol 2,4-cyclodiphosphate synthase; HDS: 4-hydroxy-3-methylbut-2-enyl diphosphate synthase; HDR: 4-hydroxy-3-methylbut-2-enyl diphosphate reductase; GGPPS: geranylgeranyl diphosphate synthase; PSY: phytoene synthase; PDS: phytoene desaturase; ZISO: ζ-carotene isomerase; ZDS: ζ-carotene desaturase; CRISO: carotenoid isomerase; LCYB: lycopene β-cyclase; CYP97: heme-containing cytochrome P450 enzymes; VDE: violaxanthin deepoxidase; ZEP: zeoxanthin epoxidase; VDL1: violaxanthin deepoxidase-like protein 1; ZEP1: zeoxanthin epoxidase 1; IPP: isopentenyl diphosphate; DMAPP: dimethylallyl diphosphate; FPP: farnesyl diphosphate; GPP: geranyl diphosphate; GGPP: geranylgeranyl diphosphate. Dashed arrows: biosynthetic processes not yet fully elucidated.

Fucoxanthin, an oxygenated isoprenoid compound belonging to the carotenoid class, has emerged as a highly valued active compound due to its remarkable efficacy in anticancer activity, fatty liver mitigation, sugar metabolism regulation, and lipid reduction [84]. Currently, the chemical synthesis of fucoxanthin has not yet been successfully achieved, nor has a comprehensive understanding of its complete biosynthetic pathway been established (Figure 3) [83]. Notably, the fucoxanthin content in *P. tricornutum* (Table 1) is significantly higher than that in common commercial sources (approximately 0.7–2.1 mg/g of dry cell weight (DCW)) [84], which are mainly derived from macroalgae. Additionally, *P. tricornutum* harbors a well-characterized genome that facilitates the systematic dissection of the molecular regulatory network and key functional genes in the fucoxanthin biosynthetic pathway. Coupled with its mature genetic engineering tools, this enables precise manipulation: overexpressing rate-limiting enzymes to direct carbon flux toward fucoxanthin synthesis, knocking out redundant pathways that compete for precursor metabolites, and optimizing promoter and terminator elements to fine-tune gene expression levels. These applications directly contribute to the elevation of fucoxanthin accumulation. Collectively, these advantageous attributes make P. tricornutum a promising microbial chassis for efficient fucoxanthin production.

Isopentenyl diphosphate (IPP) and dimethylallyl diphosphate (DMAPP) are the primary precursors for fucoxanthin synthesis. Therefore, increasing the levels of IPP and DMAPP is an effective strategy to boost fucoxanthin production. Notably, *P. tricornutum* possesses two distinct pathways for IPP and DMAPP biosynthesis: the 2-C-methylerythritol 4-phosphate (MEP) pathway in the chloroplast and the mevalonate (MVA) pathway in the cytosol [58]. Overexpression of 1-deoxy-D-xylulose 5-phosphate synthase (DXS), 4-diphosphocytidyl-2-C-methyl-D-erythritol kinase (CMK) and 2-C-methyl-d-erythritol 4-phosphate cytidylyltransferase (CMS) in the MEP pathway has been confirmed as an effective method to enhance fucoxanthin production [69,70]. However, the role of the cytosolic MVA pathway in fucoxanthin synthesis remains poorly understood to date. Studies in *Arabidopsis thaliana* have shown that shared metabolites of the MEP and MVS pathways, such as IPP or DMAPP, can translocate to the cytosol to sustain sterol biosynthesis when the MVA pathway is impaired [85]. While the regulation mechanisms underlying the crosstalk between the MEP and MVA pathways are still unclear, this crosstalk may represent another promising target for improving fucoxanthin production in *P. tricornutum*.

Three IPP molecules and one DMAPP molecule are condensed by geranylgeranyl diphosphate synthase (GGPPS) to form GGPP. Two GGPP molecules then undergo condensation catalyzed by phytoene synthase (PSY) to produce phytoene, which is subsequently converted to β-carotene via a series of sequential reactions. In algae, this process typically involves five key enzymes: phytoene desaturase (PDS), ζ-carotene isomerase (ZISO), ζ-carotene desaturase (ZDS), carotenoid isomerase (CRTISO), and lycopene β-cyclase (LCYB) [86]. All these genes have been identified in *P. tricornutum* [86], with the functions of PSY, PDS, ZDS, CRTISO, and LCYB experimentally validated [71,87,88]. Notably, this process in bacteria and fungi begins with a CrtI-type phytoene dehydrogenase, which catalyzes a four-step continuous dehydrogenation to form all-trans-lycopene. β-carotene is then synthesized from all-trans-lycopene: in bacteria, this reaction is catalyzed by lycopene cyclase (CrtY), while in fungi, it is catalyzed by phytoene-β-carotene synthase (CrtYB) [89]. Fewer enzymes in bacteria and fungi are required to synthesize β-carotene, representing an alternative strategy to heterologously express this pathway in *P. tricornutum* to enhance fucoxanthin production.

The biosynthesis pathway of fucoxanthin from β-carotene has not yet been fully elucidated. Previous studies have confirmed that violaxanthin and neoxanthin, two β-carotene derivatives, serve as intermediates in fucoxanthin biosynthesis [88,90]. Dinoxanthin, another carotenoid, was also identified as a direct precursor of fucoxanthin [91]. However, the biosynthetic route from neoxanthin to dinoxanthin or fucoxanthin has long remained elusive. In *P. tricornutum*, β-carotene undergoes hydroxylation at both terminals, which is catalyzed by the heme-containing cytochrome P450 enzymes (CYP97) to form the xanthophyll zeaxanthin [92]. Zeaxanthin and violaxanthin are interconvertible via the catalytic action of zeaxanthin epoxidase (ZEP) and violaxanthin deepoxidase (VDE). Additionally, violaxanthin can be converted to neoxanthin by the action of a violaxanthin deepoxidase-like protein 1 (VDL1) [93]. Furthermore, VDL2 catalyzes the tautomerization of diadinoxanthin to allenoxanthin [91]. Besides the violaxanthin cycle, *P. tricornutum* also harbors a diadinoxanthin cycle, which presumably relies on ZEP and VDE for the interconversion between diadinoxanthin and diatoxanthin. Notably, isoforms of ZEP exhibit distinct functional specificities: ZEP2/3 are implicated in the conversion of zeaxanthin to violaxanthin and diadinoxanthin to diatoxanthin, respectively, while ZEP1 is responsible for the conversion of haptoxanthin to phaneroxanthin [91]. A key insight into the terminal step of fucoxanthin biosynthesis comes from studies on mutants deficient in CRTISO5, a carotenoid isomerase-like protein. This mutant accumulates phaneroxanthin and is completely devoid of fucoxanthin. This finding not only indicates that CRTISO5 catalyzes the final step of fucoxanthin biosynthesis but also contradicts the previous notion that dinoxanthin acts as an intermediate in the fucoxanthin biosynthetic pathway in *P. tricornutum* [94]. Although the complete synthetic pathway of fucoxanthin remains elusive, several studies, such as those focusing on the overexpression of VDL1 [57,72] and ZEP3 [73], have confirmed that such genetic manipulation is an effective strategy to enhance fucoxanthin production in *P. tricornutum*.

Beyond direct pathway engineering, plastid lipid-associated protein (PAP) [74] and transcriptional regulators, including mediator subunit (MED8) [95], heat shock factor (HSF1) [96], and cryptochrome [75], have been shown to modulate the expression of carotenogenic genes, thereby further fine-tuning fucoxanthin production. These modifications, which employ diverse approaches to increase the yield of fucoxanthin and other carotenoids, represent promising targets for the construction of high-value carotenoid cell factories. Notably, fucoxanthin is synthesized in plastids and forms the fucoxanthin–chlorophyll a/c–protein complex (FCP) with chlorophyll a/c and antenna proteins, which localizes to the thylakoid membrane [97]. The stable chlorophyll/carotenoid molar ratio emphasizes the coordination between chlorophyll and fucoxanthin accumulation [62]. Thus, achieving fucoxanthin overproduction requires consideration of its storage form, such as concurrently increasing other components of the FCPs.

### 4.2. Eicosapentaenoic Acid (EPA)

EPA can effectively enhance cardiovascular function, alleviate depression, and reduce blood pressure in humans [98,99]. Compared to traditional fish oil, the conventional source of EPA, algae-derived EPA, has a simpler composition and is more easily extracted from cells. Thus, it serves as an excellent source for food supplements, health products, and pharmaceuticals [81]. *P. tricornutum* is well recognized for its ability to accumulate intracellular EPA, with its content accounting for up to 35% of total fatty acids [76,77]. In this species, most EPA accumulates in polar lipids, particularly galactolipids such as monogalactosyldiacylglycerol (MGDG) and digalactosyldiacylglycerol (DGDG); it is also present in some triacylglycerol (TAG) species, especially under nitrogen stress [100,101].

**Figure 4:**
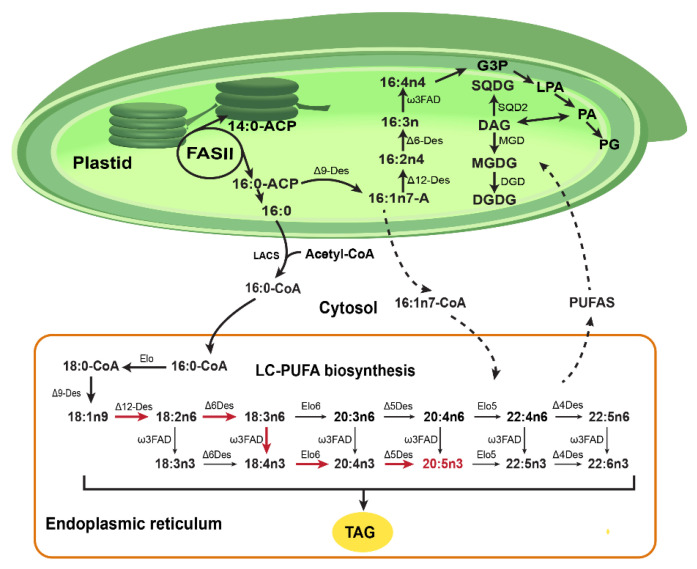
Biosynthetic pathway of polyunsaturated fatty acids in *P. tricornutum*. Red arrows: the predominant route of EPA biosynthesis in *P. tricornutum*. Dashed arrows: possible fatty acid transport routes between the ER and the chloroplast. FASII: type II fatty acid synthase; ACP: acyl carrier protein; Elo: elongase; Des: desaturase; FAD: fatty acid desaturase; LACS: long-chain acyl-CoA synthetase; DGDG: digalactosyldiacylglycerol; MGDG: monogalactosyldiacylglycerol; DAG: diacylglycerols; SQDG: sulfoquinovosyldiacylglycerol; DGD: Digalactosyldiacylglycerol synthase; MGD: Monogalactosyldiacylglycerol synthase; SQD2: Sulfoquinovosyldiacylglycerol synthase 2; G3P: glycerol-3-phosphate; LPA: lysophosphatidic acid; PA: phosphatidic acid; PG: phosphatidylglycerol; LC-PUFA: long-chain polyunsaturated fatty acid; TAG: triacylglycerol.

In terms of common mechanisms, *de novo* fatty acid synthesis in *P. tricornutum* occurs in plastids, where type II fatty acid synthase generates C16:0-acyl carrier protein (ACP) using acetyl-CoA and NADPH as the two main substrates [101]. These acyl-ACPs can be channeled into two metabolic pathways (Figure 4) [76]. First, they can undergo desaturation within the plastid: C16:0-ACP is converted to C16:1 n7 by Δ9 acyl-ACP desaturase and further sequentially desaturated by different soluble ACP desaturases (Δ12-desaturase, Δ6-desaturase, and ω3-desaturase) to form C16:2 n4, C16:3 n4, and C16:4 n1, respectively [101]. These fatty acids are then incorporated into plastidial membrane lipids. Second, acyl chains can be cleaved from ACP by thioesterase, producing free fatty acids such as C16:0 and C16:1n7, which are subsequently transported to the cytosol. Although the mechanism underlying plastid membrane crossing remains unclear, current models propose that fatty acids are exported to the cytosol [101]. There, free fatty acids are converted to acyl-CoA by long-chain acyl-CoA synthetase (LACS) and then transported to the endoplasmic reticulum (ER) via an as-yet-unidentified mechanism. Imported acyl-CoA can either be directly incorporated into glycerolipids or undergo elongation and desaturation to generate long and very long-chain polyunsaturated fatty acids, such as EPA (C22:5 n3). It has been shown that EPA can be synthesized in *P. tricornutum* by a number of different routes, with the predominant route proceeding via Δ6-desaturation of linoleic acid (Figure 4, red arrows) [77]. Once EPA is synthesized in the ER, further transport back to the plastid occurs for EPA chains, as this fatty acid is mostly found in plastidial galactolipids [100].

To date, extensive efforts have been devoted to boosting EPA production in *P. tricornutum*, including three key strategies: first, enhancing the supply of fatty acid substrates (e.g., overexpressing malonyl CoA-acyl carrier protein transacylase [78]); second, reconstructing the competitive pathway (e.g., disrupting acyl-ACP Δ9-desaturase [76]); and third, strengthening the EPA synthetic pathway (e.g., overexpression of Δ5-fatty acid desaturase gene [79] and Δ6-fatty acid desaturase gene [80]). *P. tricornutum* accumulates a large proportion of its biomass as neutral lipids, predominantly TAG, which possess suitable properties for biodiesel conversion [101]. Therefore, redirecting more EPA toward TAG incorporation (rather than shuttling it back to the plastid for galactolipid synthesis) could serve as an alternative strategy to enhance EPA production.

### 4.3. Heterologous Products

Beyond its native metabolites, *P. tricornutum* has also been engineered as a cell factory for the production of heterologous compounds, with terpenoids serving as a typical example [58,102,103,104]. The majority of terpenoids are synthesized from the universal precursors dimethylallyl pyrophosphate (DMAPP) and isopentenyl pyrophosphate (IPP). In silico predictions have indicated that genetic engineering of industrial microorganisms to coexpress both pathways can increase the theoretical maximum yield for terpenoid precursors [105]. Yoon et al. introduced a heterologous MVA pathway into *E. coli* to significantly boost IPP and DMAPP supply, achieving a β-carotene titer of 465 mg/L [106]. Notably, *P. tricornutum* naturally produces these two terpenoid precursors via two distinct metabolic pathways: the MEP localized in the chloroplast and the MVA pathway in the cytosol, representing a significant advantage. This inherent dual-pathway system avoids complex, time-consuming genetic engineering to introduce exogenous pathways for boosting IPP and DMAPP, as each such pathway requires coexpression of over six genes (Figure 4). Instead, only targeted modulation of the rate-limiting steps in the MVA and MEP pathways, respectively, enables precursor efficient accumulation, thereby establishing *P. tricornutum* as a promising chassis for terpenoid production.

In addition to its potential for terpenoids, *P. tricornutum* has emerged as a promising microalgal cell factory for bioplastic production, particularly poly-3-hydroxybutyrate (PHB) [15,107]. It harbors substantial intracellular acetyl-CoA pools, which serve as the direct metabolic precursor for PHB biosynthesis via a three-step enzymatic cascade, with reported yields reaching up to 10.6% of dry cell weight [15]. Beyond bioplastics, *P. tricornutum* has also been exploited for the production of therapeutic recombinant proteins, such as monoclonal antibodies (mAbs) [108,109]. To date, full-length mAbs have been efficiently expressed in this host [16,109], with demonstrated N-glycosylation on their Fc moiety and robust binding affinity to human Fcγ receptors [110]. Notably, mAbs produced in *P. tricornutum* exclusively carry oligomannoside N-glycans that are found on mammalian proteins, thus avoiding potential immunogenicity in humans [111]. Collectively, these data highlight the suitability of diatom-derived antibodies for human therapeutic applications.

## 5. Cultivation of *P. tricornutum*

### 5.1. Cultivation Systems

A variety of cultivation systems are applicable for large-scale propagation of microalgae, including outdoor open ponds with different designs (circular and raceway) and various types of photobioreactors (PBRs) that can be used either indoors or outdoors, such as tubular (vertical, horizontal, and helical), flat-plate, and fermenter. Currently, *P. tricornutum* can be cultivated in deep tanks, open ponds, and photobioreactors (PBRs). Notably, PBRs have recently received increasing attention as the focus of indoor or outdoor research for the industrial cultivation of *P. tricornutum* (Table 2). According to a statistical analysis by Pang et al. [84], indoor cultivation of *P. tricornutum* generally yields higher biomass and fucoxanthin production than outdoor cultivation, with average increases of 2-fold and 3-fold, respectively. Buono et al. [112] evaluated the biomass productivity, carbon dioxide fixation rate, and biochemical composition of *P. tricornutum* in indoor high-technology photobioreactors (HT-PBRs) and outdoor systems (both pilot ponds and low-technology photobioreactors in a greenhouse in southern Italy). Their findings showed that the biomass productivity of *P. tricornutum* in 1/2 SWES standard medium was approximately doubled in HT-PRB compared to open ponds. However, although outdoor open ponds exhibit lower biomass productivity than indoor PBRs, PBRs are more costly. To reduce the cost of microalgal biomass, it is imperative to develop a large-scale cultivation system characterized by high biomass productivity, low production and downstream processing costs, and ease of operation.

### 5.2. Culture Media

To date, the F/2 medium and its derivatives remain the most widely used culture media for *P. tricornutum* [84,126]. Specifically, nitrogen-enriched F/2 formulations (with nitrogen concentrations adjusted to 2–25 times the standard F/2 level) have been extensively employed in research [113,126], consistently achieving higher fucoxanthin accumulation compared to non-nitrogen-supplemented F/2 media. This optimization strategy yields an average fucoxanthin content of 27 mg/g dry cell weight, with a maximum recorded value of 59.2 ± 22.8 mg/g dry cell weight [113].

As a core macronutrient, nitrogen significantly regulates the growth performance and metabolite biosynthesis of *P. tricornutum*. A variety of nitrogen sources (e.g., nitrate, ammonium, and urea) have been thoroughly evaluated for suitability in cultivating *P. tricornutum*. Among these, urea outperforms other single or mixed nitrogen sources in promoting both biomass accumulation and the biosynthesis of fucoxanthin and fatty acid [127]. Notably, nitrogen limitation is a well-established strategy for lipid production in diatoms, as it redirects cellular carbon flux from protein synthesis toward lipid biosynthesis. Compared with nitrate, urea possesses a key advantage: it does not accumulate as unassimilated nitrogen in the culture medium, thereby avoiding misjudgments of nitrogen sufficiency in large-scale cultivation systems [128].

Phosphorus is another key nutrient involved in cellular energy transfer and the biosynthesis of multiple cellular components (e.g., nucleic acids and phospholipids). *P. tricornutum* can utilize diverse phosphorus sources, with the inorganic form (Pi, PO4^3−^) being more rapidly and efficiently assimilated compared to dissolved organic phosphorus (DOP) [129]. Under phosphorus limitation, biomolecule production in diatoms is enhanced during the transition from the logarithmic to the stationary phase. In this state, cell division eventually ceases, and a larger proportion of carbon flow is redirected toward triacylglycerol accumulation as storage compounds or excretion as extracellular polysaccharides (EPS) [130]. Conversely, increased phosphorus concentrations in the medium correlate with elevated cell division rates [129].

Generally, nitrogen or phosphorus is the primary limiting nutrient for *P. tricornutum* growth, as other minerals required for its proliferation are typically present in sufficient quantities. Alterations in nutrient supply are usually reflected in their relative ratios, and the calculated N:P ratio can predict the growth characteristics and metabolic product profiles of this diatom. Li et al. [131] found that the growth rate of *P. tricornutum* reached its maximum at an N:P ratio of 16:1 and decreased significantly when the ratio was either higher or lower than 16:1. Thus, when the N:P ratio exceeds 16:1, phosphorous becomes the limiting factor for organic carbon production; conversely, an N:P ratio below 16:1 indicates nitrogen limitation [131,132,133].

Silicate is a crucial nutrient for the cell wall biosynthesis in most diatoms; however, it is non-essential for *P. tricornutum*, as this species possesses both silicified (oval form) and non-silicified (fusiform and triradiate forms) organic cell wall morphotypes. Consistent with this characteristic, recent studies have demonstrated that supplementation with various concentrations of sodium metasilicate does not affect the growth or fatty acid profile of *P. tricornutum* [128]. Overall, silicate availability exerts minimal influence on the growth of *P. tricornutum* compared to nitrogen and phosphorus.

CO_2_ serves as the primary inorganic carbon source for the photosynthetic growth of *P. tricornutum*. Elevated CO_2_ concentrations have been shown to enhance both the growth rate and lipid productivity of *P. tricornutum* [134,135]. Notably, only dissolved CO_2_ is bioavailable to microalgae, as the speciation of inorganic carbon (i.e., CO_2_, H_2_CO_3_, HCO^3−^ and CO3^2−^) in the liquid phase is predominantly governed by the medium pH [136]. However, CO_2_ sparging induces a reduction in medium pH. Specifically, in a large-scale cultivation system, sparging with >5% CO_2_ (*v*/*v*) results in a solution pH of 5.6–6.5 [64]. A previous study has demonstrated that pH fluctuations between 6.5 and 9.5 have no significant impact on the maximal specific growth rate of *P. tricornutum*, while cultures maintained at pH < 6.5 or >9.5 exhibit substantially reduced growth performance [64].

In addition to inorganic carbon, most *P. tricornutum* strains are capable of utilizing organic carbon sources (e.g., glycerol, acetate, glucose, fructose and lactic acid) under mixotrophic mode [137]. Among these substrates, glycerol has been predominantly selected as the optimal supplement in most studies [138]. Compared to autotrophic culture, mixotrophy promotes cell growth and biomass accumulation but concomitantly reduces photosynthesis activity, leading to a decrease in photosynthetic pigment content [139]. Fucoxanthin, as a key photosynthetic pigment, is thus negatively affected by this trade-off. Consequently, achieving simultaneous high biomass yield and high fucoxanthin content in mixotrophically grown *P. tricornutum* remains a major challenge. The development of an efficient mixotrophic cultivation strategy is therefore critical for the commercial-scale production of fucoxanthin.

### 5.3. Temperature

Temperature is a crucial environmental factor that profoundly regulates the growth and metabolism of marine diatoms. *P. tricornutum* is considered a eurythermal species, capable of thriving within a broad temperature range of 5–30 °C [140]. The optimal growth temperature for *P. tricornutum* is generally recognized to be approximately 20 °C, with suboptimal growth observed at temperatures exceeding 25 °C or below 15 °C [141]. However, certain tropical strains (e.g., Pt9_F28 °C_) have been reported to exhibit enhanced growth rates at temperatures above 25 °C [142]. Recent studies have indicated that heat shock transcription factors (HSFs) play a potential key role in thermal tolerance [96,143], and overexpression of PtHSF2 can enhance the tolerance of *P. tricornutum* to 30 °C [143]. In general, metabolic rates increase with rising temperatures until reaching an optimal threshold; further temperature elevation can adversely affect metabolic rates, impairing processes such as photosynthesis and growth. However, for the biosynthesis of specific high-value compounds (e.g., EPA), modified temperature conditions often prove beneficial, as they promote metabolic flux toward the accumulation of target products [96,144].

### 5.4. Light

Light is the major energy source for photosynthesis, and the absorption of different light intensities and qualities can alter the growth and fucoxanthin production of *P. tricornutum* [145]. Previous studies have documented increased growth rates under high irradiance: for instance, Chrismadha and Borowitzka [146] observed this phenomenon at 56 to 1712 μmol photons m^−2^ s^−1^, and Nogueira et al. [147] reported similar results at 50, 300 and 600 μmol photons m^−2^ s^−1^. Conversely, photosynthetic pigments (e.g., chlorophyll a, chlorophyll c, and fucoxanthin) as well as PUFAs (e.g., EPA) show significant accumulation at relatively low irradiance levels. The photosynthetic capacity of diatoms depends on a specific range of light intensities, which may provide different mechanisms and strategies for optimizing the photosynthetic rates to maximize the utilization of available photons [148].

Diatoms are exposed not only to fluctuations of light intensity but also to changes in light quality. Specifically, red light promotes cell growth, while blue light enhances fucoxanthin accumulation [149]. Notably, the intracellular fucoxanthin content under green light is comparable to that observed under white light conditions. In response to these findings, two cultivation strategies—mixed blue and red light and the two-phase culture with dynamic light regime adjustment—have been developed to simultaneously enhance biomass and fucoxanthin production [28,149]. For example, a two-phase culture system was established by shifting the red: blue light ratio from 6:1 (phase 1, biomass accumulation stage) to 5:1 (phase 2, fucoxanthin synthesis stage), achieving a final biomass of 6.52 g/L and a fucoxanthin productivity of 8.22 mg/L/d [28]. Additionally, compared to red-blue LED light and white fluorescent light, single blue light-emitting diode (LED) illumination reduces energy input by 50% and 75%, respectively, which confers significant advantages for large-scale industrial applications [150].

## 6. Current Challenges and Perspectives

*P. tricornutum* exhibits considerable potential as a chassis for sustainable bioproduction, yet it remains a niche product within the algal industry. In Europe, for instance, its annual production volume was estimated to be a mere 4 tonnes [151]. Currently, large-scale cultivation of *P. tricornutum* primarily relies on autotrophic systems, which typically yield biomass concentrations below 3 g/L [116]. In contrast, mixotrophic cultivation can markedly boost biomass productivity; however, it has not been adopted for industrial-scale production due to key technological bottlenecks, including a high contamination risk associated with organic nutrient supplementation in open cultivation systems [152] and considerable production costs incurred when using photobioreactors [115]. Furthermore, while mixotrophic culture outperforms autotrophic culture in terms of cell growth rate and biomass accumulation, it often compromises photosynthetic activity, ultimately leading to reduced contents of photosynthetic pigments (one of the most valuable products in *P. tricornutum*) [139]. Thus, elucidating the molecular mechanisms underlying pigment biosynthesis and its regulation networks, followed by genetic modification of P. tricornutum to enhance target product accumulation under mixotrophic cultivation, holds great promise for advancing its commercial-scale application. In addition, heterotrophic modification of *P. tricornutum* can significantly enhance its biomass accumulation [30,31]. Accordingly, balancing biomass production and target product synthesis in *P. tricornutum* by genetic engineering, followed by heterotrophic fermentation, represents an alternative strategy for its industrial adoption.

To date, the metabolic profile and predicted intrinsic metabolic networks of *P. tricornutum* have been constructed primarily based on integrated omics data [153,154]. Nevertheless, biosynthesis pathways such as that of fucoxanthin encompass numerous enzymatic reactions, with the characterization of both the complete biosynthesis pathway and its rate-limiting steps remaining inadequate. Currently, flow cytometry [155] and fluorescence-activated cell sorting (FACS) [60] have been employed for strain screening in *P. tricornutum*, based on its fluorescence intensity. These high-throughput screening methods enable the acquisition of phenotypically diverse strains, thereby providing more opportunities to identify the complete biosynthesis pathway and deepen understanding of the species’ metabolic behavior. However, relying solely on spontaneous fluorescence is insufficient for sorting phenotypically diverse strains; additional methods such as Raman-activated cell sorting (RACS) [156] and high-throughput mass spectrometry [157] thus require further development.

As a photoautotrophic chassis cell, *P. tricornutum* holds substantial application potential. However, endowing it with competitive advantages and realizing large-scale production may not be merely by modifying one or a few genes. Currently, technical challenges persist in site-specific gene expression and continue multi-site editing, highlighting the need for efficient, precise gene editing techniques (e.g., multi-gene editing and seamless/traceless gene editing technologies). To date, CRISPR/Cas9-mediated homology-directed insertion has been successfully applied for antibiotic resistance gene insertion in *P. tricornutum* [91], but the proportion of correct transformants remains significantly lower than that of false positives. A previous study reported that knockdown of a DNA ligase IV homolog (*ligIV*) in *P. tricornutum* effectively enhances homologous recombination (HR) efficiency [49]. Meanwhile, knockout of KU70/KU80, key genes in the nonhomologous end joining (NHEJ) pathway, has been confirmed to improve HR efficiency in organisms such as *Yarrowia lipolytica* [158] and *Ganoderma lucidum* [159]. Accordingly, a modified strain with high HR efficiency may be constructed, which could be further used as the chassis for cell factories.

In summary, the diatom *P. tricornutum* stands out as a distinctive photosynthetic chassis for synthetic biology, boasting a fully sequenced and well-annotated genome, mature gene editing toolkits, the capacity to synthesize high-value metabolites, comprehensively characterized phenotypic traits, and inherent scalability for industrial deployment. However, compared to other well-established synthetic biology chassis, such as *E. coli* and *S. cerevisiae*, this algal strain faces critical bottlenecks that demand urgent resolution for industrial large-scale application, with persistently high product costs being the core limitation. In addition to optimizing algal growth traits and enhancing biomass accumulation efficiency and target product yields via genetic engineering, industrial-scale precision-controlled cultivation designed for stable, high-quality production of *P. tricornutum*-derived products also serves as a key pathway to overcoming these bottlenecks. Particularly against the backdrop of the rapid development of China’s green power industry, the high-value development and utilization of *P. tricornutum* is emerging as a highly promising route for the efficient conversion of green power resources. Therefore, technological breakthroughs should be advanced through a two-pronged strategy: (1) improving the photosynthetic efficiency and intracellular carbon fixation efficiency of algal strains via biotechnological approaches while enhancing their robustness for large-scale cultivation and their product synthesis capacity, and (2) upgrading the electricity–light–biomass conversion efficiency of cultivation systems through engineering methods, followed by developing an adaptive large-scale production system integrated with green power application scenarios to form a technical closed loop and accelerate industrialization.

## Figures and Tables

**Table 1:** Strain improvement strategies of *P. tricornutum*.

Strains	Strategies	Culture Conditions	Light	Products	Content/Increase	Refs
*P. tricornutum* (CCAP 1055/1)	Random mutagenesis using UV and strain screening by FACS	ASW medium; 21.0 °C	24:0 h; 150 µmol/m^2^/s;	Fx	<1.10-fold of WT	[60]
	Random mutagenesis using EMS and strain screening by FACS	ASW medium; 21.0 °C	24:0 h; 150 µmol/m^2^/s;	Fx	1.35-fold of WT	
	Random mutagenesis using X-ray and strain screening by FACS	ASW medium; 21.0 °C	24:0 h; 150 µmol/m^2^/s;	Fx	<1.10-fold of WT	
*P. tricornutum* (CCAP 1055/1)	Random mutagenesis using EMS	f/2 medium; 22.0 ± 2.0 °C	24:0 h; 30 µmol/m^2^/s;	Fx	1.69-fold of WT	[62]
*P. tricornutum* (CCAP 1055/1)	Random mutagenesis using UV	Modified f/2 medium; 22.0 ± 2.0 °C	24:0 h; 25 µmol/m^2^/s;	Fx	2.7-fold of WT	[61]
	Photo-oxidative stress-driven mutagenesis and ALE	Modified f/2 medium; 22.0 ± 2.0 °C	24:0 h; 25 µmol/m^2^/s;	Fx	2.1-fold of WT	
*P. tricornutum* (CCAP 1055/1)					2.1-fold of WT at pH 5.5	[64]
	Acidic stress-based ALE	f/2 medium; 22.0 ± 2.0 °C	-; 100 ± 5 µmol/m^2^/s;	Growth rate	1.46-fold of WT at pH 6.0	
					1.27-fold of WT at pH 6.5	
*P. tricornutum* UTEX 646	WT	ASP-mdeium; 18.0 ± 1.0 °C	16:8 h; 40 µmol/m^2^/s;	Fx	10.30 ± 0.50 mg/g;	[69]
	Overexpression of *dxs*	ASP-mdeium; 18.0 ± 1.0 °C	16:8 h; 40 µmol/m^2^/s;	Fx	24.20 ± 0.20 mg/g; 2.40-fold of WT	
	Overexpression of *psy*	ASP-mdeium; 18.0 ± 1.0 °C	16:8 h; 40 µmol/m^2^/s;	Fx	18.40 ± 1.50 mg/g; 1.80-fold of WT	
*P. tricornutum* (CCMP 2561)	Overexpression of *cmk*	Modified f/2 medium; 20.0 ± 0.5 °C	12:12 h; 100 µmol/m^2^/s;	Fx	21.69 mg/g; 1.83-fold of WT	[70]
	Overexpression of *cms*	Modified f/2 medium; 20.0 ± 0.5 °C	12:12 h; 100 µmol/m^2^/s;	Fx	21.51 mg/g; 1.82-fold of WT	
*P. tricornutum* (NRIA-0065)	Overexpression of *psy*	f/2 medium; 20.0 °C	12:12 h; 90 µmol/m^2^/s;	Fx	1.45-fold of WT	[71]
*P. tricornutum* (CCMP632)	Overexpression of *PtVDL1*	ASW medium; 20.0 °C	12:12 h; 100 µmol/m^2^/s;	Fx	1.15-fold of WT	[72]
*P. tricornutum* (CCMP2561)	Coexpression of *Vde*, *Vdr, Zep3*	f/2 medium; 18.0 °C	12:12 h; 90 µmol/m^2^/s;	Fx	0.135 ± 0.004 pg/cell; 4.00-fold of WT	[73]
*P. tricornutum* (MACC/B228)	Overexpression of *PAP*	Modified f/2 medium; 24.0 °C	-; 80 µmol/m^2^/s;	Fx	1.33-fold of WT	[74]
				EPA	24.50 ± 0.13%/TFA	
*P. tricornutum*	Knockout of *CryP*	-; 20.0 °C	12:12 h; 80 µmol/m^2^/s;	Fx	Increase	[75]
*P. tricornutum* UTEX 646	Knockout of acyl-ACP Δ9 desaturase	f/2 medium; 20.0 °C	-; 100 µmol/m^2^/s;	EPA	1.32–1.42 -fold of WT	[76]
*P. tricornutum* UTEX 646	Overexpression of *OtElo5*	ESAW medium; 20.0 °C	24 h; 60 µmol/m^2^/s;	DHA	8.00-fold of WT	[77]
*P. tricornutum* (CCMP 2561)	Coexpression of *MCAT* and *PtD5b*	f/2 medium; -	24 h; -	DHA	9.15 μg/mg	[78]
				EPA	85.35 μg/mg	
*P. tricornutum* (CCMP 2561)	Overexpression of *PtD5b*	f/2 medium; 21.0 ± 0.5 °C	15:9 h; -	EPA	1.58-fold of WT	[79]
*P. tricornutum* (LAMB014)	Overexpression of Δ6 FAD	f/2 medium; 22.0 ± 1.0 °C	12:12 h; 100 µmol/m^2^/s;	EPA	38.101 mg/g; 1.48-fold of WT	[80]

WT: wild type of P. tricornutum; Fx: fucoxanthin; TFA: total fatty acids; -: no data.

**Table 2:** Cultivation techniques of *P. tricornutum*.

Strains	Culture Method	Working VOL	Light Quality	Light Intensity	Light: Dark	Culture Cycle/Day	Cell Biomass /Productivity	Fx Content/Maximum Productivity	EPA Content/Maximum Productivity	Refs
*P. tricornutum* CS-29	Indoor, flat panel PBR	5 L	White LED light	150 μmol/m^2^/s	12:12 h	13 day	0.17–0.59 g/L	59.20 ± 22.80 mg/g DCW; 20.50 mg/d/L	-	[113]
*P. tricornutum*	Indoor, vertical flat-plate glass PBR	240 cm length, 120 cm height and 6 cm width	-	70–100 μmol/m^2^/s	-	-	2.35 ± 0.12 g/L	1.33 ± 0.03% DCW	4.42 ± 0.06% DCW	[114]
*P. tricornutum*	Indoor, flat panel airlift PBR	180 L	LED	8 μmol/m^2^/s	-	21 day	1.53 g/L/d	16.30 mg/g DCW	40.50 mg/g DCW	[115]
*P. tricornutum*	Indoor, tubular PBR	400 L	Uncontrolled condition	Uncontrolled condition	Uncontrolled condition	-	-	2.50 mg/d/L	-	[116]
*P. tricornutum*	Indoor, plastic cylinders	30 L	60 W fluorescent lamps	37.5 μmol/m^2^/s	-	11 day	-	7.14 mg/g DCW	-	[117]
*P. tricornutum* UTEX 646	Indoor, hanging bag PBR	20 L	White light	Step1: 120 μmol/m^2^/s; Step2: 30 μmol/m^2^/s;	-	15 day	1.30 g/L	8.32 mg/L	62.55 mg/L	[118]
*P. tricornutum* Mi136.M1.a	Indoor, PBR, semi-continuous	5000 L	Natural light	-	-	-	-	13.30 ± 0.30 mg/g DCW	29.10 ± 1.30 mg/g DCW	[119]
*P. tricornutum* SAG 1090-1a	Indoor, HT-PBR, semi-continuous	250 L	58 W fluorescent lamps	174 μmol/m^2^/s	-	-	21.00 ± 2.30 g/m^2^/d	-	-	[112]
	Outdoor, LT-PBR,	100 L	-	-	-	-	4.90 ± 2.00 g/m^2^/d	-	-	
	Outdoor, open ponds	4.6 × 2.4 m	-	-	-	-	10.90 ± 3.70 g/m^2^/d	-	-	
*P. tricornutum* CS-29	Indoor, bubble column RBR	50 L	LED light	360/μmol/m^2^/s	16: 8 h	12 day	160 mg/L/d	-	-	[120]
*P. tricornutum* N017	Outdoor, tubular PBR	800 L	-	-	-	30–48 days	1.50–2.50 g/L	0.40% DCW	-	[121]
*P. tricornutum*	Outdoor, bubble column PBR	200 L	Natural light	-	-	14 day	0.96 ± 0.04 kg/m^3^;	8.55 ± 0.56 mg/g	-	[122]
*P. tricornutum*	Outdoor, horizontal tubular PRB	2000 L	Natural light	-	-	-	-	1.67 ± 0.14 mg/g	-	[123]
*P. tricornutum* PTN0301	Indoor, column PBR	70 L	cool white neon	300/μmol/m^2^/s	16: 8 h	24 day	-	-	32.88 ± 3.22%/TFA	[124]
	Outdoor, vertical column PBR, semi-continuous	270 L	-	-	-	27 day	0.30–1.00 g/L	-	36.43 ± 0.99%/TFA	
	Outdoor, open ponds, semi-continuous	1000 L	-	-	-	32 day	0.30–0.80 g/L	-	22.73 ± 2.19%/TFA	
*P. tricornutum* UTEX 640	Outdoor, pyrex glass tube PBR	51 L				14 day	1.00 g/L	-	10% of the ash-free DCW	[125]
	Outdoor, circular pond	1 m diameter, 30 cm height				14 day	0.30–0.60 g/L	-	9% of the ash-free DCW	

DCW: dry cell weight; -: no data.

## Data Availability

No new data were created or analyzed in this study.
